# The Cdk5 inhibitor olomoucine promotes corneal debridement wound closure in vivo

**Published:** 2008-03-17

**Authors:** Brajendra K. Tripathi, Mary A. Stepp, Chun Y. Gao, Peggy S. Zelenka

**Affiliations:** 1 Laboratory of Molecular and Developmental Biology, National Eye Institute, National Institutes of Health, Bethesda, MD; 2 Department of Anatomy, George Washington University Medical Center, Washington, DC

## Abstract

**Purpose:**

To investigate the effect of the Cdk5 inhibitor olomoucine on corneal debridement wound healing in vivo.

**Methods:**

Corneal debridement wounds of 1.5 mm were made on the ocular surface of CD-1 mice. A 20 μl drop of 15 µM olomoucine in 1% DMSO was applied to the wound area immediately after wounding and again after 6 h. Control mice received identical applications of 1% DMSO. Mice were euthanized after 18 h, two weeks, and three weeks for evaluation of wound healing and restratification. Corneas were stained with Richardson’s dye, photographed, and processed for histology and immunofluorescence as whole mounts or paraffin sections. The remaining wound area at 18 h was measured by image analysis. Scratch wounded cultures of human corneal-limbal epithelial cells (HCLE) were used to examine the effect of olomoucine on matrix metalloproteinase (MMP) expression in vitro. MMP-2 and MMP-9 were detected by immunofluorescence and immunoblotting.

**Results:**

Olomoucine treatment significantly enhanced corneal wound closure without increasing inflammation or infiltration of polymorphonuclear leukocytes 18 h after wounding (p<0.05). The increased localization of MMP-9 within epithelial cells at the wound edge was further enhanced by olomoucine while the expression of MMP-2 was reduced. Olomoucine treatment of scratch wounded HCLE cells produced similar changes in MMP-9 and MMP-2 expression. The examination of treated corneas two and three weeks after wounding showed normal epithelial restratification with no evidence of inflammation or stromal disorganization.

**Conclusions:**

Topical application of olomoucine in 1% DMSO significantly enhances closure of small epithelial debridement wounds without increasing inflammation or impairing reepithelialization.

## Introduction

Cells along the leading edge of corneal debridement wounds undergo specific changes in gene expression, cytoskeletal organization, and signaling that enable them to maintain tight connections with neighboring cells while migrating rapidly to cover the wound [1]. Among the changes observed in these cells is a specific activation of the Ser/Thr kinase, Cdk5 [2]. Cdk5 activity in this region was shown to limit the accumulation of active Src at the plasma membrane [2]. Active Src stimulates the formation of lamellipodia and the dynamic turnover of cell-cell junctions thus promoting epithelial cell migration [3]. However, excessive Src activity can also cause degradation of E-cadherin [4] and a complete loss of cell-cell adhesion, leading to epithelial-to-mesenchymal transition (EMT) [5], so its activity and localization must be stringently controlled. By limiting the accumulation of active Src along the leading edge, Cdk5 protects the integrity of the epithelial cell sheet but somewhat reduces the rate of cell migration. Thus, inhibiting Cdk5 activity in organ culture after debridement wounding enhances the formation of lamellipodia and significantly increases the rate of migration but also causes some separation of cells along the leading edge [2]. Conversely, the overexpression of Cdk5 in corneal epithelial cells of transgenic mice significantly reduces the rate of debridement wound closure [2]. The ability of Cdk5 inhibitors to increase epithelial cell migration during wound closure in organ culture suggested that the pharmacological manipulation of Cdk5 activity might be therapeutically useful in some situations if it did not interfere with cell-cell adhesion or produce other detrimental effects [6]. In this study, we examine the feasibility of this approach by testing the ability of the Cdk5 inhibitor, olomoucine, to promote closure of small corneal epithelial debridement wounds in mice.

## Methods

### Corneal debridement

All procedures conformed to the guidelines provided by the Association for Research in Vision and Ophthalmology and the National Institutes of Health, Bethesda, MD. Six-week-old male CD-1 mice (approximately 20 g) were purchased from Charles River Laboratories (Wilmington, MA) and housed under standard laboratory conditions; water and food were continuously available. Animals were anesthetized with a mixture of ketamine (70 mg/kg), xylazine (7 mg/kg), and acepromazine (10 mg/kg). Small (1.5 mm) corneal debridement wounds were made as previously described with minor modifications [7]. One group of mice was euthanized immediately after wounding (t=0 h) for measurements of the initial wound area. The remaining mice were divided into treatment and control groups. The treatment group received 15 μM olomoucine (Sigma, Indianapolis, IN), which was prepared in phosphate buffered saline (PBS; Invitrogen, Carlsbad, CA) containing 1% DMSO. The control group received 1% DMSO in phosphate buffered saline. Olomoucine was applied twice (at 0 h and 6 h) as a single drop (20 μl) to the central cornea of the injured eye with the lower eyelid held away from the eye to avoid overflow. Both groups received erythromycin ophthalmic ointment to keep the cornea moist and to prevent infection.

**Figure 1:**
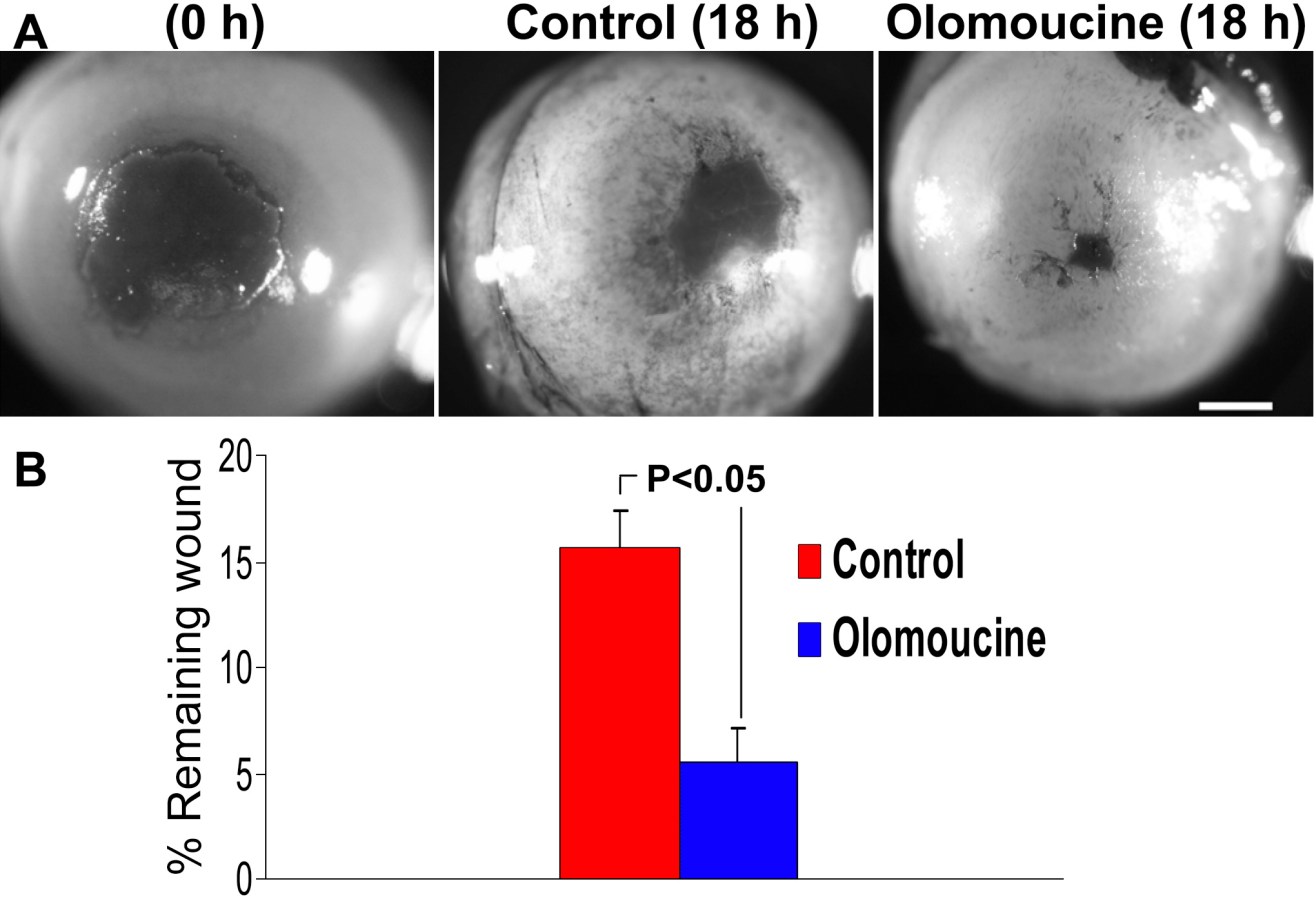
Olomoucine treatment promotes corneal re-epithelialization in normal mice. **A**: Eyes were stained with Richardson’s stain immediately after debridement wounding (0 h) or 18 h afterward. At 0 h and 6 h after wounding, control animals received a sterile vehicle (1.0% DMSO in PBS) and the treatment group received 15 μM olomoucine. Scale bar=100 μM. **B**: The graph shows the remaining wound area as a percentage of average initial wound area (mean ± SEM). The difference between olomoucine-treated and control mice was statistically significant (p<0.05; n=28–30).

**Figure 2:**
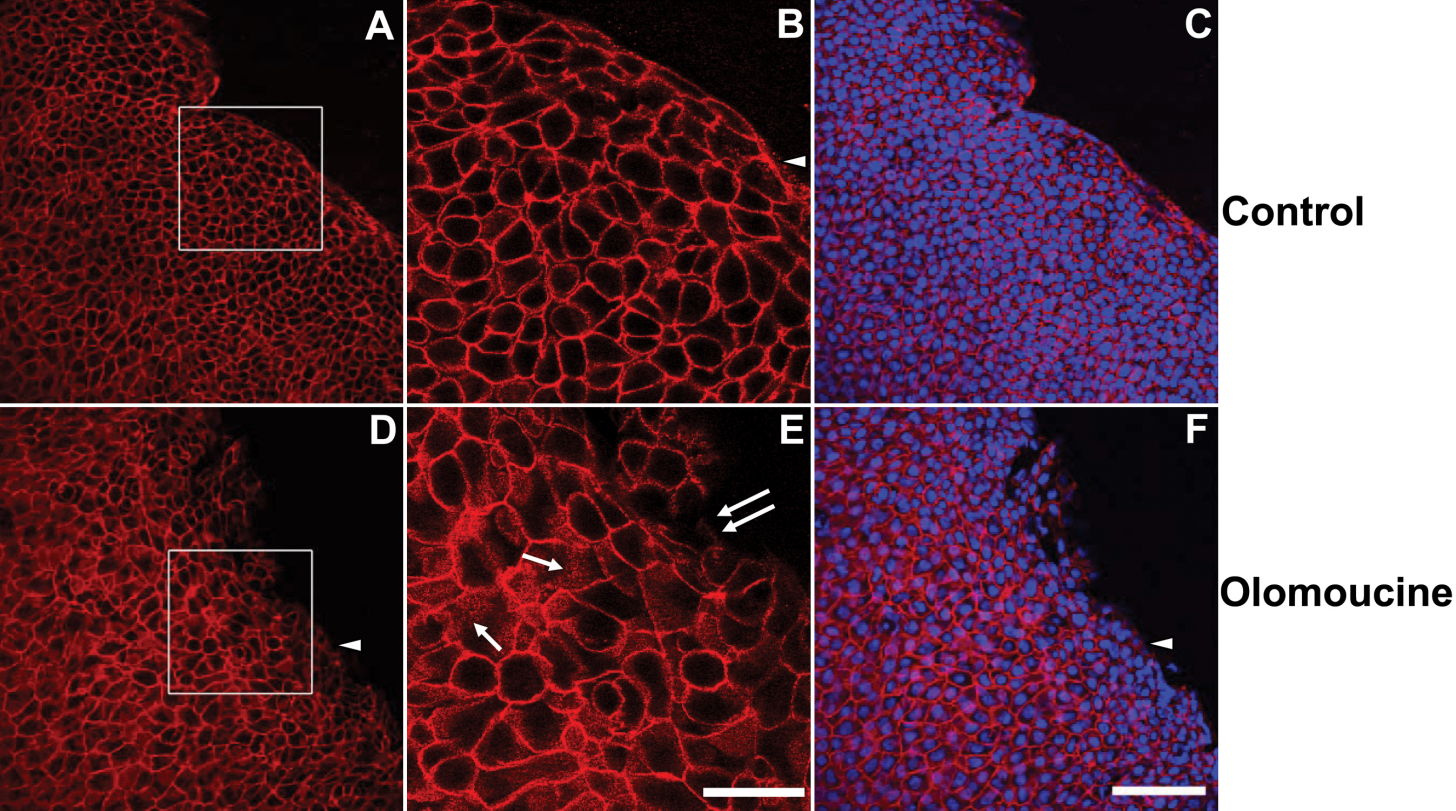
Olomoucine treatment does not disrupt the integrity of the epithelial cell sheet. **A**: The immunofluorescence of E-cadherin in epithelial cells of corneal whole mounts of control animals shows tightly packed epithelial cells with a smooth, advancing cell front. Cells were compact and cell density was high especially along the wound edge. **B**: Higher magnification of the boxed area shown in panel **A** shows that E-cadherin immunofluorescence in control corneas was confined almost entirely to cell-to-cell boundaries. Punctate E-cadherin staining was seen at the migrating front on the edge lacking cell-to-cell contacts (arrowhead). **C**: Superimposition of E-cadherin immunostaining and DAPI-staining of nuclei confirms that E-cadherin is located at cell-to-cell boundaries and that all cells express E-cadherin. **D**: In olomoucine-treated corneas, cell density was appreciably lower and the migrating front was irregular. Cell-to-cell junctions appeared to be intact except for a few cells at the wound edge. Immunostaining of E-cadherin was weak or undetectable at the migrating front on the edge lacking cell-to-cell contacts (arrowhead). **E**: Higher magnification of the boxed area shown in panel **D** demonstrates punctate intracellular immunostaining for E-cadherin in many cells (single arrows). E-cadherin localization at cell-to-cell junctions was disrupted in a few cells along the wound edge (double arrows). **F**: Superimposition of E-cadherin immunofluorescence and DAPI-staining of nuclei confirms that E-cadherin is located at cell-to-cell boundaries and demonstrates that all cells express E-cadherin. Scale bar=100 μM in **A**,**C**,**D**, **F**; 40 μM in **B**,**E**.

### Histological analysis

For photography of corneal abrasions, animals were euthanized 18 h, two weeks, and three weeks after wounding. Eyes were removed, fixed with 4% paraformaldehyde, and stained with Richardson’s dye. Photomicrographs were taken using an Olympus dissecting microscope (Olympus, Center Valley, PA), and the remaining wound area was measured by image analysis using Image ProPlus (Media Cybernetics, Pleasanton, CA). Images were identified only by code until measurements were completed to avoid experimenter bias. For sectioning, enucleated eyes were fixed for 24 h in 4% paraformaldehyde (for paraffin sections) or 10% formalin (for methacrylate sections) and embedded accordingly. Corneal sections (6 μm) were stained with hematoxylin and eosin and evaluated by light microscopy.

### Cell culture

Human corneal limbal epithelial (HCLE) cells were grown to confluency at 37 °C in a humidified atmosphere of 95% air and 5% CO_2_ in Keratinocyte-SFM medium (Invitrogen) supplied with pre-qualified human recombinant epidermal growth factor 1–53 (EGF 1–53), and bovine pituitary extract (BPE). The medium was supplemented with 0.3 mM calcium chloride (Invitrogen) and 50 µg/ml penicillin/streptomycin.

### Scratch wounding and immunoblotting

For in vitro analysis of protein expression, HCLE cells were seeded onto 60 mm dishes coated with a fibronectin-collagen coating mixture (Biologic Research Faculty & Facility, Ijamsville, MD), grown to 90% confluence, and scratch wounded by multiple linear scrapes with a sterile 20 μl pipette tip. After washing away debris, the cells were re-fed with the medium. Olomoucine (15 μM) was added to experimental samples for 6 h immediately after wounding. Control samples were treated identically except that olomoucine was omitted. After 6 h of treatment, cells were lysed in PBSTDS buffer (1X phosphate buffered saline, 1% Triton X-100, 0.5% sodium deoxycholate, 0.1% SDS containing Complete™ protease and phosphatase inhibitor), and immunoblotting was performed as described previously [8].

### Antibodies

Anti-MMP-2 and anti-MMP-9 polyclonal antibodies were purchased from Abcam Inc., (Cambridge, MA). Anti-E-cadherin polyclonal antibody was purchased from Cell Signaling Technology (Beverly, MA). Alexa 488-conjugated and Alexa 568-conjugated anti-rabbit IgG and DAPI were purchased from Molecular Probes (Eugene, OR).

### Immunofluorescence and confocal microscopy of whole mount cornea

After treatment, eyes were removed, fixed, rehydrated and stained with anti-MMP-2, anti-MMP-9 and anti-E-cadherin antibodies according to previously described protocol [9]. After staining, the corneal tissues were removed and washed in PBS and whole-mounted on Superfrost microscope slides (Erie Scientific Company, Portsmouth, NH) for laser scanning confocal microscopy (Leica TCS SP2, Leica Microsystem, Germany) as previously described [10].

**Figure 3:**
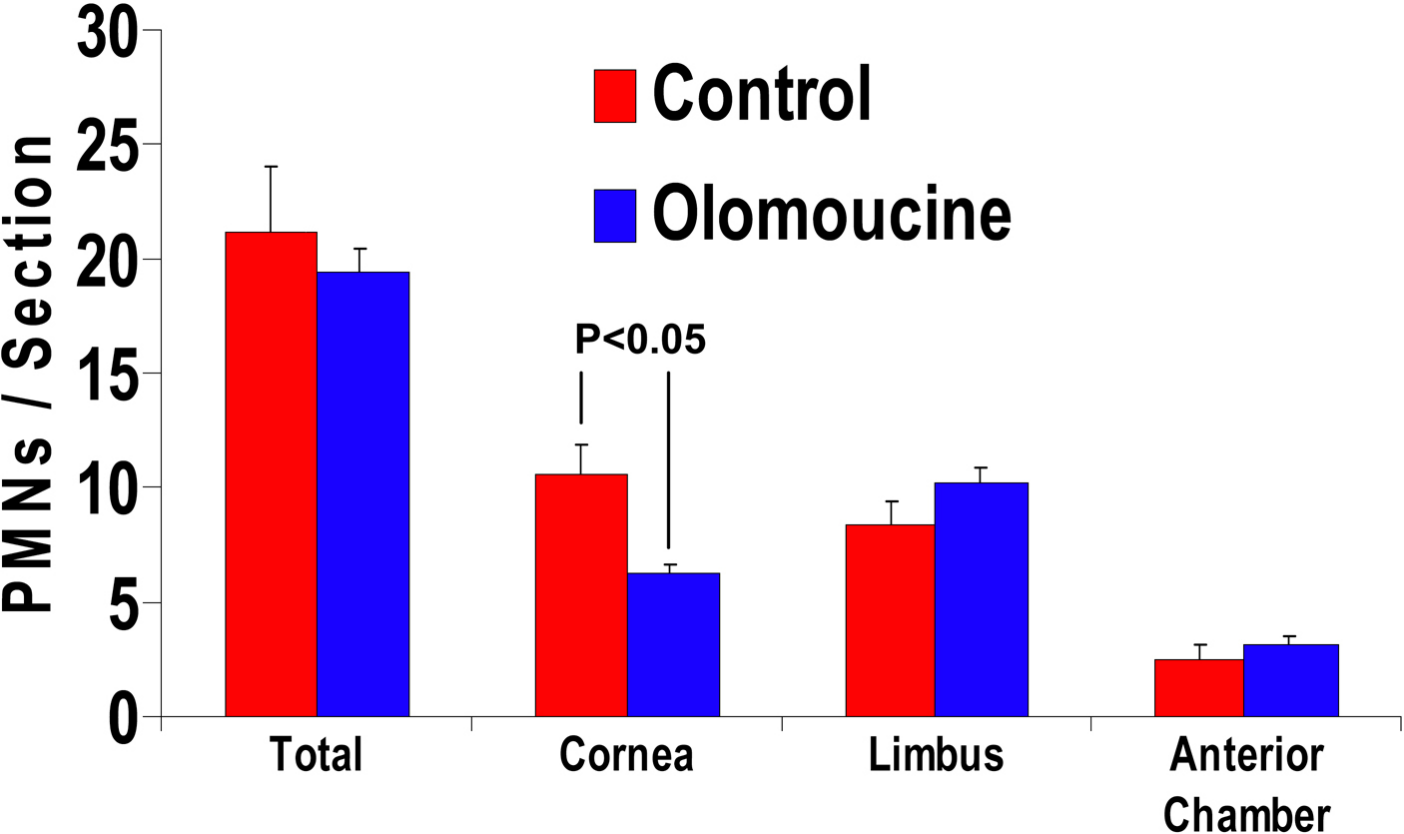
Olomoucine does not increase infiltration of polymorphonuclear neutrophils (PMNs) 18 h after debridement wounding. The counting of PMNs in the photomicrograph of corneal sections stained with hematoxylin/eosin showed no significant difference in the average total number of PMNs per section. The examination of specific regions indicated a significant reduction in the number of PMNs in the central cornea (p<0.05; n=10), which was compensated by small increases in the limbus and anterior chamber.

**Figure 4:**
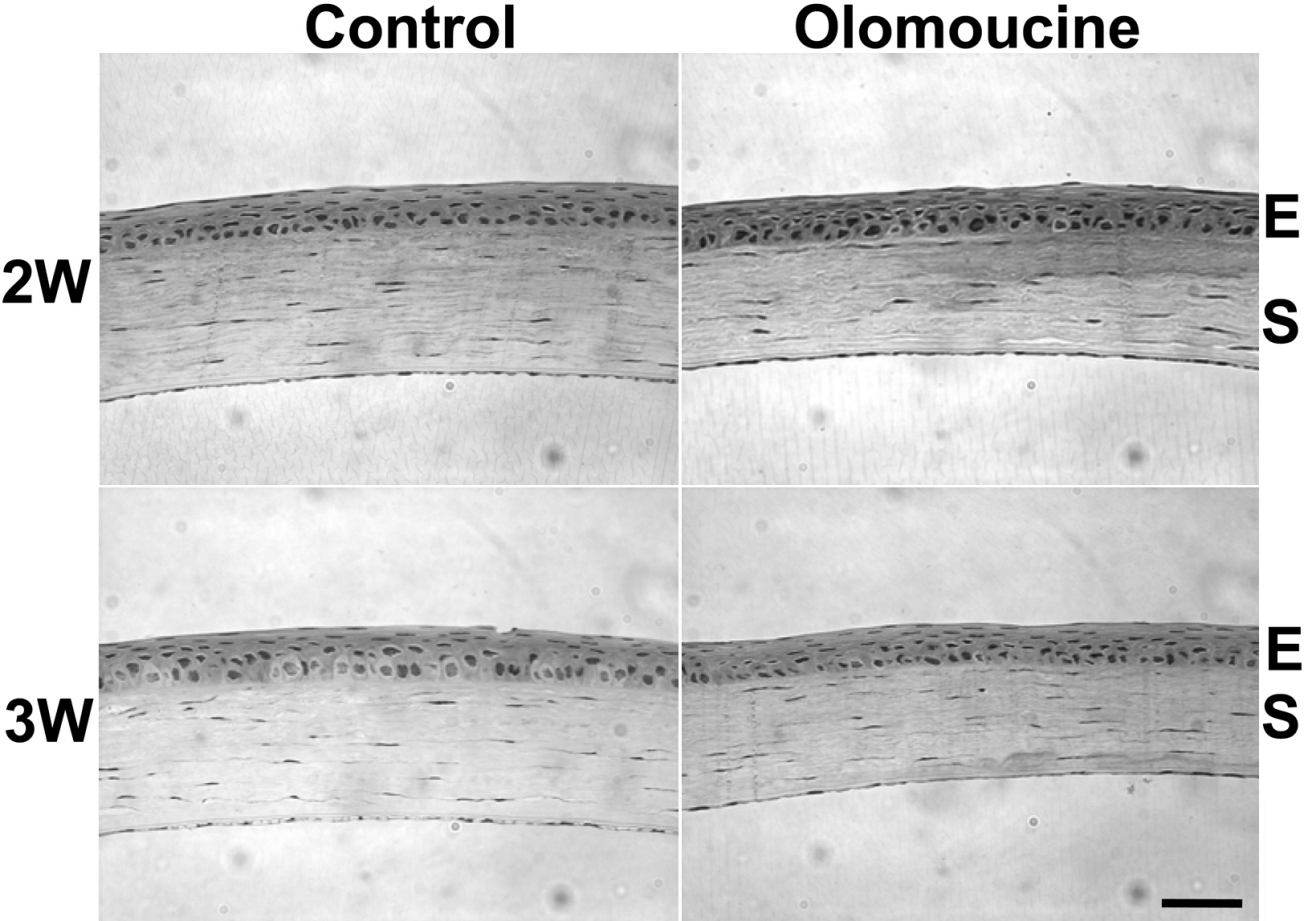
Olomoucine treatment supported normal restratification of the corneal epithelium. Photomicrographs of hematoxylin/eosin stained sections of the restratified wound area of olomoucine-treated and untreated control corneas two and three weeks after corneal abrasion revealed no apparent differences in epithelial morphology or stromal organization. W=week; E=epithelium; S=stroma. Scale bar=100 μM.

### Immunohistochemical staining of MMP-2 and MMP-9 in paraffin sections

Formalin-fixed, paraffin-embedded sections (6 μm) were deparaffinized with xylene and rehydrated through a graded alcohol series. Samples were permeabilized in 0.25% Triton X-100 in PBS for 10 min and incubated with 5% normal goat serum in PBS for 2 h to block non-specific sites. Sections were incubated overnight at 4 °C with the primary antibody at a dilution of 1:150 (V/V). Sections were extensively washed in phosphate buffered saline and then incubated with Alexa 488-conjugated or Alexa 568-conjugated anti-rabbit IgG (Molecular Probes) at a dilution of 1:250 (V/V). Sections were counterstained with DAPI at a dilution of 1:2500 in PBS (V/V) for nuclear staining and mounted. Negative controls contained no primary antibody.

### Data analysis

Data were analyzed by ImagePro Plus (MediaCybernetics, Silver Spring, MD) and/or ImageQuant (GE Healthcare, Piscataway, NJ) image analysis software. Values of all data are expressed as mean ± standard error mean. For statistical analysis of data sets, Student’s *t*-test was performed using SigmaPlot (Systat, San Jose, CA) software and a p<0.05 was considered statistically significant.

## Results

### In vivo corneal wound closure

A mouse corneal debridement wound model was used to test the effect of olomoucine on corneal epithelial wound closure in vivo. The central cornea was demarcated with a 1.5 mm trephine, and debridement wounds were made without encroaching on the limbus or conjunctiva. The initial wound area varied less than 5% as determined by image analysis of the stained cornea (not shown). Mice were given two topical applications of 15 μM olomoucine in 1% DMSO, one immediately after wounding and the other 6 h later. Control animals received equivalent treatments with 1% DMSO only. Animals were euthanized after 18 h and the remaining wound area was stained, photographed, and measured by image analysis software. Images were identified only by code to avoid experimenter bias during the measurements. The results showed that the remaining wound area in olomoucine-treated corneas was significantly less than in controls, indicating that in vivo treatment with olomoucine enhances epithelial wound closure (Figure 1A,B).

### E-cadherin localization in olomoucine-treated corneas

Since previous studies had suggested that inhibition of Cdk5 activity might also affect E-cadherin junctional stability, we examined E-cadherin localization in treated and untreated corneas 18 h after wounding. In control corneas, E-cadherin immunostaining was confined almost entirely to cell-cell boundaries (Figure 2). Only cells at the leading edge, which lacked cell contacts on one surface, showed appreciable cytoplasmic staining of E-cadherin. Cells were compact and cell density was high especially along the wound edge. In contrast, in olomoucine-treated corneas, cells were more spread and cell density was appreciably lower. Cell-cell junctions appeared to be intact for the most part, although many cells contained E-cadherin positive vesicles (Figure 2). Small disruptions in the migrating front suggest there may be some loss of cell-cell adhesion in this region, but it is not widespread.

**Figure 5:**
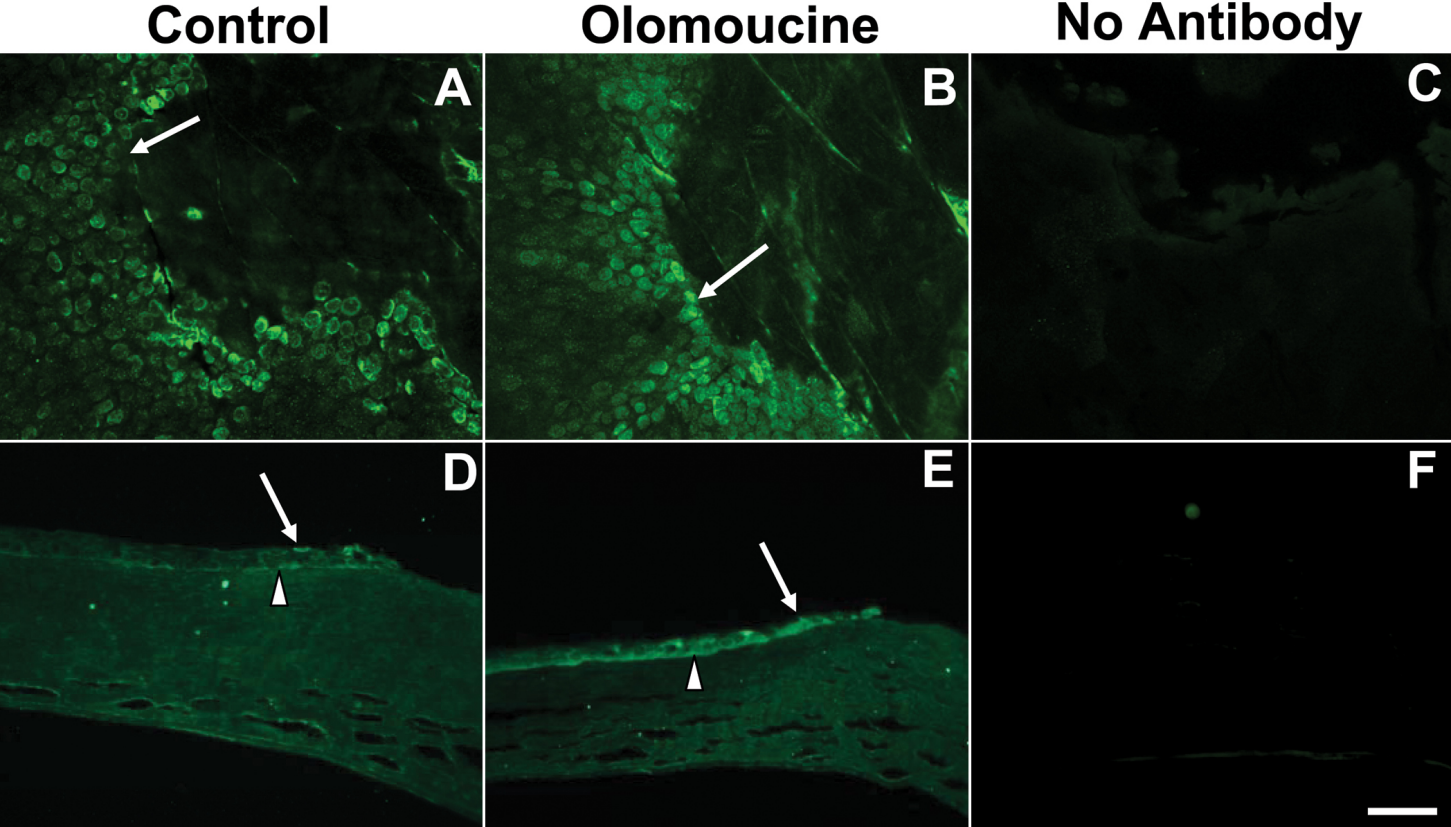
Olomoucine treatment increased the localization of MMP-9 protein in epithelial cells at the wound edge. **A**: MMP-9 immunofluorescence is elevated at the wound edge in untreated control corneas (arrow). **B**: The immunostaining of MMP-9 at the wound edge (arrow) is further enhanced in olomoucine-treated corneas. **C**: Whole mounted corneas without any primary antibody showed no immunofluorescence. **D**: Paraffin sections of wounded control corneas showed MMP-9 immunofluorescence in all layers of the cornea at wound edge (arrow) and in basal epithelial cells distal to the wound (arrowhead). **E**: Paraffin section of olomoucine-treated corneas showed intense MMP-9 immunofluorescence in a wider band of cells at the wound edge. Immunofluorescence was seen in cells of all layers of the epithelium (arrow). **F**: No immunostaining was detected in the paraffin sections of corneas when primary MMP-9 antibody was omitted. Scale bar=100 μM.

**Figure 6:**
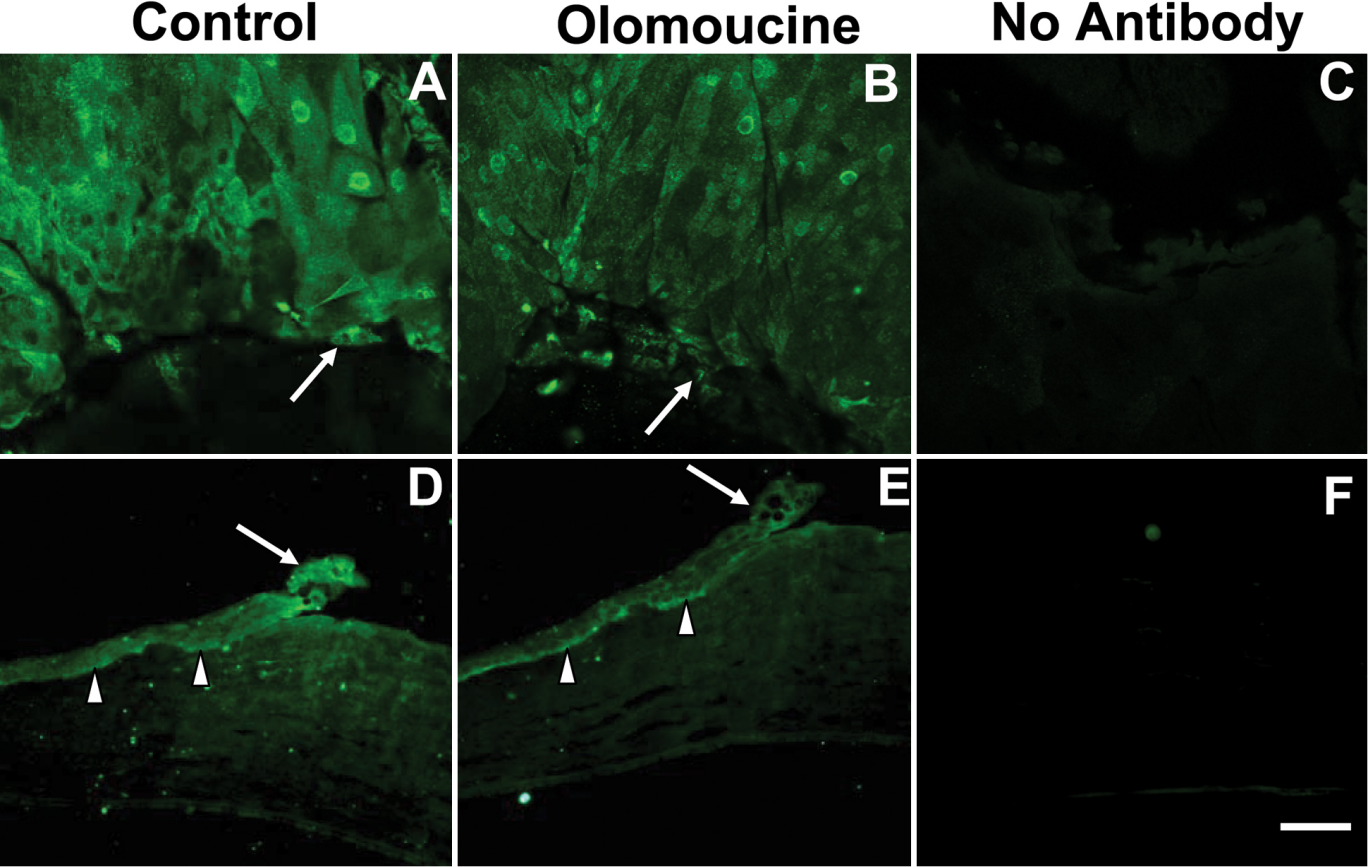
Olomoucine treatment decreased localization of MMP-2 protein at the wound edge. **A**: MMP-2 immunofluorescence is elevated at the wound edge in untreated control corneas. **B**: The immunostaining of MMP-2 at the wound edge (arrow) is reduced in olomoucine-treated corneas. **C**: Whole mounted corneas without primary antibody showed no immunofluorescence. **D**: Paraffin sections of wounded control corneas showed MMP-2 immunofluorescence in all layers of the cornea at wound edge (arrow) and in basal epithelial cells distal to the wound (arrowhead). **E**: The paraffin section of olomoucine-treated cornea showed reduced immunofluorescence of MMP-2 at the wound edge (arrow). MMP-2 immunofluorescence persisted in the basal layers of epithelium (arrowhead). **F**: No immunostaining was detected in the paraffin section of the cornea when primary MMP-2 antibody was omitted. Scale bar=100 μM.

### Polymorphonuclear leukocyte (PMN) infiltration

To determine whether olomoucine treatment affects neutrophil infiltration, we counted the number of polymorphonuclear leukocytes (PMNs) present in the limbal stroma, corneal stroma, and anterior chamber of hematoxylin/eosin stained methacrylate sections of olomoucine-treated and control eyes. PMNs were identified by nuclear morphology, and two sections from each of the 10 eyes were used in each group. As before, sections were identified only by code to avoid experimenter bias. The results showed that PMN infiltration of the corneal stroma was significantly less in olomoucine-treated eyes (p<0.05). In contrast, the number of PMNs in the limbal stroma and anterior chamber of treated eyes was not significantly different from controls (Figure 3).

### Restratification

To assess the long-term effects of olomoucine treatment on restratification of the corneal surface, we examined the corneal histology of seven paraffin sections from each of the five treated and five untreated eyes two and three weeks after wounding. We observed no difference in epithelial thickness or number of cell layers within the original wound area of olomoucine-treated corneas when compared to untreated controls. Epithelial cells appeared to adhere well to each other and to their basement membrane. Treated corneas also showed no abnormalities in basement membrane thickness, stromal organization, or keratocyte number (Figure 4).

### Matrix metalloproteinase expression

Since matrix metalloproteinases play an important role in corneal epithelial cell migration [11], we next determined whether inhibition of Cdk5 by olomoucine also affected MMP expression by examining corneal whole-mounts and paraffin sections, which were immunostained with antibodies specific for MMP-9 and MMP-2. Untreated controls showed increased expression of MMP-9 in several rows of cells surrounding the wound margin as previously reported [12]. MMP-9 expression was seen primarily on the basal aspect of basal cells. In the olomoucine-treated eyes, the immunostaining of MMP-9 appeared more intense and more cells were positively stained in both the whole mounts and paraffin sections (Figure 5).

It is known that MMP-2 (gelatinase A) participates in epithelial repair and stromal remodeling [12]. To determine whether olomoucine affects the expression of MMP-2 in the wounded corneal epithelium, whole mounted corneas and paraffin sections were immunostained for MMP-2. In contrast to the results seen for MMP-9, immunofluorescence of this matrix metalloproteinase was significantly less in corneal epithelium upon treatment with olomoucine in both the whole mounted corneas and the paraffin sections (Figure 6).

**Figure 7:**
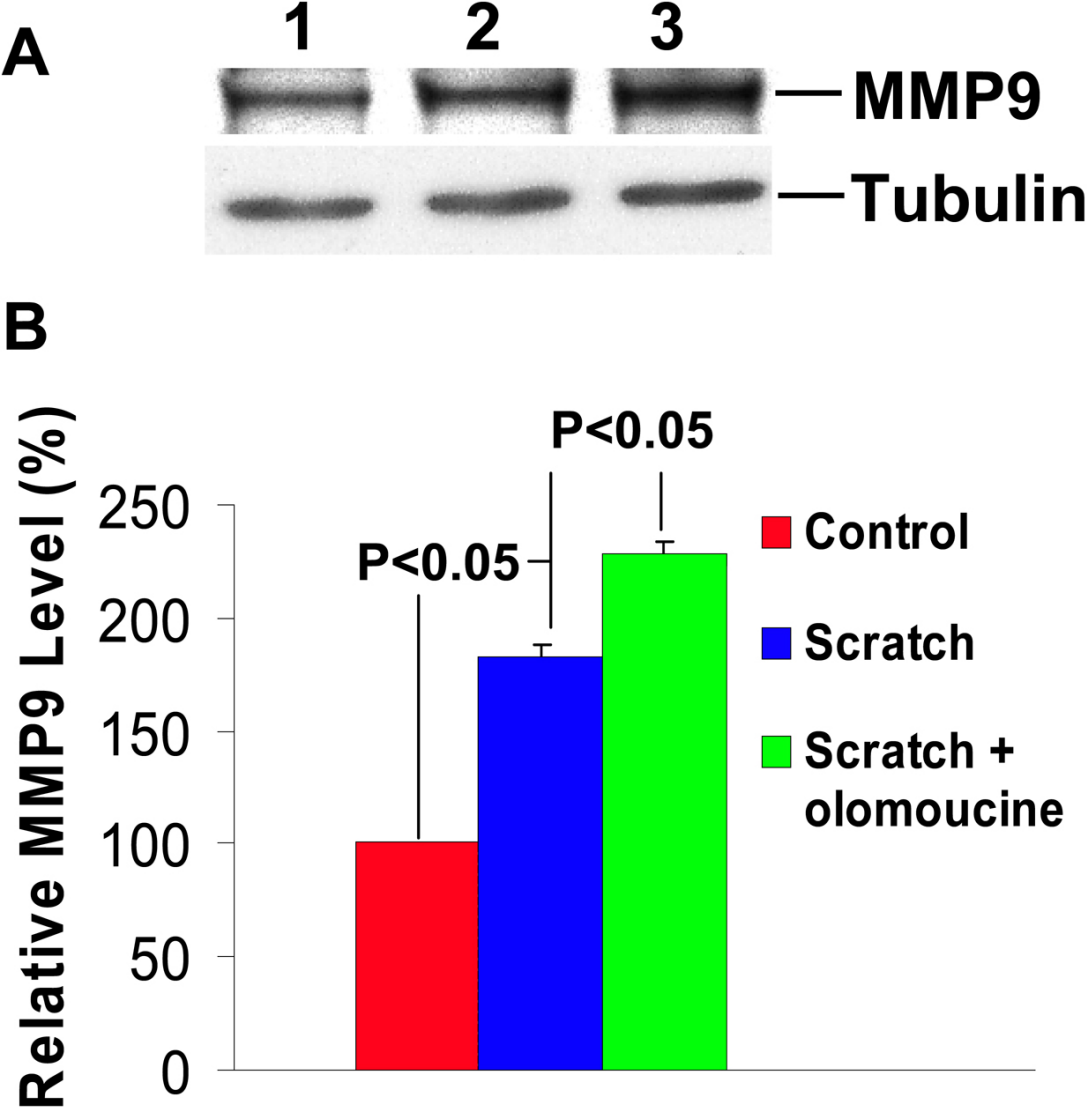
Olomoucine treatment significantly increases MMP-9 expression in HCLE cells after scratch wounding. **A**: Immunoblotting of protein extracts of HCLE cells with MMP-9 antibody detected a single immunoreactive band of approximately 92 kDa. Lane 1 shows that cultured cells expressed MMP-9 before scratch wounding. Lane 2 illustrates that scratch wounding increased MMP-9 expression detected 18 h afterward. Lane 3 shows that scratch wounding followed by 18 h incubation with olomoucine further increased MMP-9 expression. **B**: The results of immunoblotting experiments were quantified by densitometry (n=4). Statistical analysis demonstrated a significant increase in MMP-9 expression (p<0.05) in scratch wounded cultures when compared to unwounded cultures and in scratch wounded cultures with olomoucine when compared to those without it. Graph represents mean ± SEM. Values are normalized to unwounded controls.

**Figure 8:**
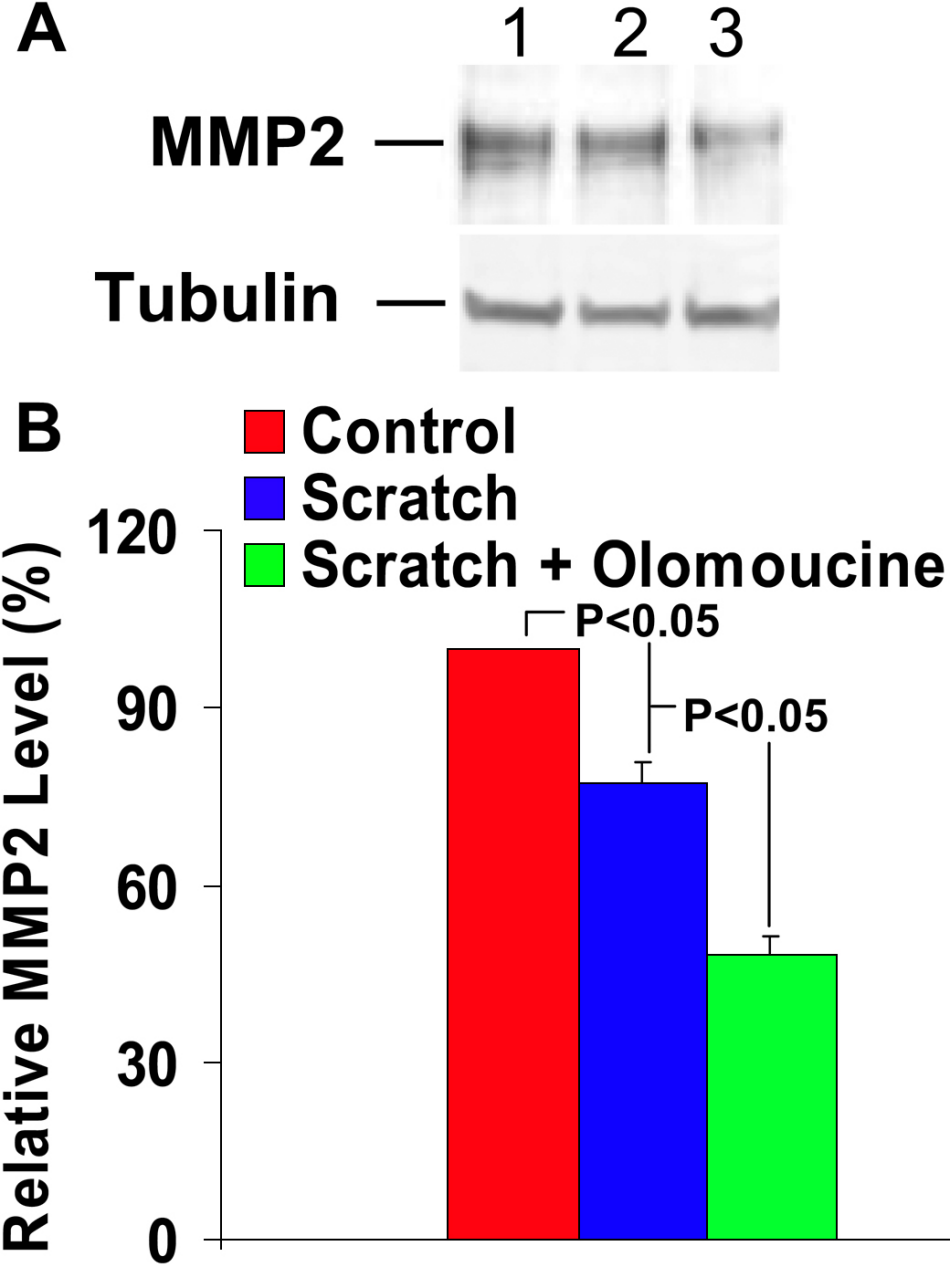
Olomoucine treatment significantly decreases MMP-2 expression in HCLE cells after scratch wounding. **A**: Immunoblotting of protein extracts of HCLE cells with MMP-2 antibody detected a single immunoreactive band of approximately 68 kDa. Lane 1 shows that cultured cells expressed MMP-2 before scratch wounding. Lane 2 illustrates that scratch wounding decreased MMP-2 expression detected 18 h afterward. Lane 3 also shows that scratch wounding followed by 18 h incubation with olomoucine further decreased MMP-2 expression. **B**: The results of immunoblotting experiments were quantified by densitometry (n=4). Statistical analysis demonstrated a significant decrease in MMP-2 expression (p<0.05) in scratch wounded cultures when compared to unwounded cultures and in scratch wounded cultures with olomoucine when compared to those without it (p<0.05). Graph represents mean ± SEM. Values are normalized to unwounded controls.

We confirmed the effect of olomoucine on MMP expression by immunoblotting proteins extracted from cultured human corneal epithelial cell line (HCLE) before and after scratch wounding to simulate the effect of corneal debridement. Immunoblotting with anti-MMP-9 antibody detected a single immunoreactive band of approximately  92 kDa that comigrated with purified MMP-9 protein, thus confirming its specificity (not shown). Scratch wounding of confluent cultures produced a significant increase in MMP-9 expression, which was further increased in the presence of olomoucine (p<0.05; Figure 7A,B). Immunoblotting also demonstrated a significant decrease in MMP-2 expression, which was further decreased by olomoucine (p<0.05; Figure 8A,B). Thus, treatment with olomoucine specifically alters the expression levels of MMP-9 and MMP-2 in corneal epithelial cells in vitro as well as in vivo.

## Discussion

The results of this study demonstrate that the topical application of olomoucine increases the rate of debridement wound closure in mice without causing appreciable dissociation or detachment of epithelial cells. While these positive findings suggest that Cdk5 inhibitors may hold promise for treating corneal erosions, some problems remain. Most importantly, all currently available Cdk5 inhibitors also significantly inhibit other members of the cyclin dependent kinase family, which are essential for cell proliferation [6,13]. However, since proliferation is not required for closure of small debridement wounds of the type used in this study [14,15], the potential inhibition of proliferation has not posed a problem. Moreover, since olomoucine is a reversible inhibitor, its effects are transient when it is applied topically, as shown by the normal restratification observed two and three weeks after wounding. These results suggest that the inhibition of Cdk5 with olomoucine or other Cdk5 inhibitors may be a useful treatment strategy for small debridement wounds; however, its therapeutic potential in other situations requires further study.

The results of this study also showed that olomoucine did not increase the infiltration of inflammatory cells into the corneal stroma as measured 18 h after wounding. Neutrophils normally promote epithelial cell proliferation and wound closure possibly by releasing growth factors stored in neutrophil granules [16,17]. However, infiltration must be strictly regulated since excessive infiltration and the ensuing inflammation are damaging [18]. The presence of normal numbers of neutrophils in treated corneas indicates that olomoucine does not disrupt the regulatory processes governing neutrophil infiltration. When regions of the cornea were examined separately, the average number of neutrophils in the central cornea was somewhat less in treated animals than in controls, while the average numbers in the limbal region and the anterior chamber were slightly more. This suggests that olomoucine may have a slight effect on the rate of infiltration, although the total number of infiltrating cells was not affected.

An additional finding of this study is the novel effect of olomoucine on matrix metalloproteinase expression. Expression of MMP-9, which is normally induced along the wound edge, was further enhanced by olomoucine treatment. Conversely, olomoucine suppressed the expression of MMP-2, which is constitutively expressed in the basal cells [19]. MMP-9 is known to play an important role in regulating corneal epithelial cell migration [11]. Previous studies have linked the induction of MMP-9 transcription along the wound edge to upregulation of Pax6, a transcription factor which binds directly to the MMP-9 promoter [20]. Since the effect of olomoucine on Pax6 expression has not yet been examined, it is not clear whether olomoucine affects this same pathway. However, there are many potential mechanisms due to the regulation of MMP-9 expression being complex and involving the concerted action of multiple other factors including AP-1, AP-2, SP-1, and NF-κB [11]. Moreover, Src plays an important role in regulating MMP transcription in many cell types by interacting with upstream signaling pathways [21-23]. Since previous studies from this laboratory have shown that olomoucine treatment increases the concentration of active Src along the wound edge [2], it will be particularly interesting to examine the possible involvement of Src family kinases in regulating MMP expression in these cells.
